# Analysis and identification of drug similarity through drug side effects and indications data

**DOI:** 10.1186/s12911-023-02133-3

**Published:** 2023-02-14

**Authors:** Amir Torab-Miandoab, Mehdi Poursheikh Asghari, Nastaran Hashemzadeh, Reza Ferdousi

**Affiliations:** 1 Department of Health Information Technology, Faculty of Management and Medical Informatics, Tabriz University of Medical Sciences, Golghast St., Tabriz, 5166614711 Iran; 2 Pharmaceutical Analysis Research Center and Faculty of Pharmacy, Tabriz University of Medical Sciences, Tabriz, Iran; 3 Research Center for Pharmaceutical Nanotechnology, Tabriz University of Medical Sciences, Tabriz, Iran

**Keywords:** Drug similarity, Similarity prediction, Indication, Side effect

## Abstract

**Background:**

The measurement of drug similarity has many potential applications for assessing drug therapy similarity, patient similarity, and the success of treatment modalities. To date, a family of computational methods has been employed to predict drug-drug similarity. Here, we announce a computational method for measuring drug-drug similarity based on drug indications and side effects.

**Methods:**

The model was applied for 2997 drugs in the side effects category and 1437 drugs in the indications category. The corresponding binary vectors were built to determine the Drug-drug similarity for each drug. Various similarity measures were conducted to discover drug-drug similarity.

**Results:**

Among the examined similarity methods, the Jaccard similarity measure was the best in overall performance results. In total, 5,521,272 potential drug pair's similarities were studied in this research. The offered model was able to predict 3,948,378 potential similarities.

**Conclusion:**

Based on these results, we propose the current method as a robust, simple, and quick approach to identifying drug similarity.

## Background

The similarity of drugs in medicine has gained significant attention in recent years because of their use in medical information processing and clinical reasoning [1]. Drug similarity trials aim to find drugs with identical pharmacological properties to the drug of interest and are motivated by the idea that similar drugs should be equivalent in the mechanism of action, have similar side effects as well as indications, and are effective in the treatment of a specific set of diseases [2, 3]. Drug-drug similarity has extensive application in various fields, such as drug repositioning, prediction of drug-drug interaction, recognition of drug target, prediction of drug side effects, and prediction of drug indications. Indications and side effects are key elements that can be used to investigate drug similarity [4, 5].

The indications based on which drugs are prescribed are valid reasons for using these drugs. One crucial problem in drug development is deducing possible new clinical targets for accepted drugs. A detailed correlation between prescription and diagnosis is not accessed straight away, although there are some freely available tools for treatment that prescribe medicine based on given indications. Such drug labels are usually called manufacturers warnings, which are then licensed by the FDA (Food and Drug Administration). Nonetheless, the use of not FDA-approved medications is widespread in clinical practice. Predicting accurate indications could significantly reduce the risk of attrition in clinical phases [6–8]. The patient's experience of diagnosis and expected symptoms provide critical information for future medical evaluation, changes in the standard of clinical care, and better support for informed decision-making. The linking and standardization of medicine and its planned uses to formal terminologies assists in managing clinical knowledge and plays an essential role in facilitating secondary use in clinical and translational research [9, 10].

Simultaneously, the drug side effect is a secondary effect of a pharmaceutical or medical treatment, which is usually unpleasant. Developing medications is a complicated process because no two individuals are precisely the same, so for certain people, developing therapies with practically no side effects may be challenging. It is also difficult to manufacture a medication that affects one part of the body but does not impact other parts, which raises the risk of side effects in the untargeted parts [11]. One important aspect of drug development is preventing side effects, including dangerous drug reactions. Another approach to detecting latent side effects of toxic medications is in vitro preclinical health screening, which measures medicines through biochemical and cellular assays. This testing approach, however, is costly and labor-intensive. Therefore, developing successful computational methods for accurately predicting medication side effects is vitally essential [12].

Measuring therapeutic drug-drug similarities quantitatively will pave a path for similarity in the prescription treatment and further analysis of patient-likeness [13]. The relationship between drug-drug can be determined from different resources. Numerous statistical methods have been successfully applied in drug-drug similarity analytics focused on product characteristics [14, 15]. Brown et al. extend methods to determine significantly co-occurring Drug-Mesh term pairs in the literature database and cluster drugs based on their pair-wise similarities [16].

Rapidly evolving techniques also allowed the processing of multiple types of drug data and thus opened up new avenues for quantitative drug discovery and drug safety studies [17, 18]. The drug similarity analysis paves the groundwork for this work as identical structural, molecular, and biological properties frequently lead to specific indications of drugs or side effects [19]. This study hypothesizes that the similarity of clinical drugs can be created from the drug indications and the drug-side effects data.

**Fig. 1:**
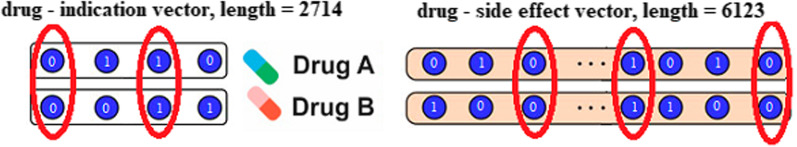
Vectors construction

Based on the systematic design methods (as shown in Fig. 1), this study compiled drug indications and drug-side effects from Side Effect Resource (SIDER 4.1) database and vectorized them. Subsequently, the drug similarity was analyzed based on indications and side effects in various ways and compared as well.

## Materials and methods

### Data extraction

The primary data source was the Side Effect Resource (SIDER 4.1) database. SIDER contains information on marketed medicines, their recorded adverse drug reactions, and their side effect frequency. In addition, SIDER includes a data set of drug indications. It provides 2997 drugs and 6123 side effects (in the drug-side effects category) as well as 1437 drugs and 2714 indications (in the drug-indications category). The information is extracted from public documents, package inserts, drug labels, off-label associations between drugs and side effects, and adverse event reporting systems that collect reports from doctors, patients, and drug companies using Natural Language Processing. The package contain information about their described drugs common and/or brand names. Based on this information, labels were mapped to STITCH 4.0. This release utilizes the MedDRA dictionary (version 16.1) and accesses preferred and lower-level terms [20]. All drug indications and drug side effects lists were extracted from SIDER for each approved drug. To generate pairs of known drug-indications and drug-side effects, each list of drug indications and side effects was extracted separately. As a result, 14,631 drug indications and 334,603 drug side effects were obtained and subjected to similarity analyses.

### Data vectorization

After data extraction, the binary vector was constructed for every approved drug. The length of the drug-indications vector was 2714. The value of each vector index was set at 1 as a positive value. The corresponding drug was associated with the related indications unless it was set at 0 as a negative value. The same procedure was performed for drug-side effects data. The length of the binary vector for drug-side effects was 6123.

### Similarity analysis

There are various methods for evaluating similarity, some of which consider only positive matches and others only negative ones. Besides, both positive and negative matching are considered in several measures [21]. Inclusion or exclusion of negative matches for a measure appears to be one of the contentious disputes in the similarity measures. Methods developed based on the inner product-based similarity measure consider only positive matches. However, for some measures, the absence of a feature in both positive and negative elements of the vector is a similarity that can be considered a negative match. Though, in other measures, the degree of variable positive/negative effect was considered [22]. Since, in this study, we want to evaluate the similarity of drugs based on their side effects as well as their indications, after building binary vectors, we employed four similarity measures that consider only positive matches to investigate the drug indications and the drug-side effects.

To evaluate measures, all the Jaccard, Dice, Tanimoto, and Ochiai similarity measures were used to estimate the similarity for drug pairs. Table 1 describes four similarity measures and their mathematical equations for measuring drug pair similarity. For each drug pair, we utilized two built vectors (i.e., side effects and indications vectors). To determine which drugs, have an important association to each other in terms of similarity of side effects and indications, similarity measures of two groups of vectors were compared to each other. We finally reached 4 similarity measures to select the best method for identifying drug similarity based on their side effects and indications. The benchmark for comparing the performance of similarity measures was the number of correct or incorrect detection and interpretation of drug indications and drug side effects for each measurement. A minimum threshold (> 0) for the carefully chosen similarity measure was set to have a discriminative standard for identifying interactions with weak or strong possibilities. In the next step, drugs with zero vector values were eliminated. Then, the similarity measures for all unknown vectors were calculated. All pairs of drugs were sorted based on their similarity measures. Finally, a list of drug pairs with high similarities in terms of indications and side effects was extracted. For the categorization of similarity or dissimilarity (based on four performance metrics), three split points were used in four categories, including: (i) low: 0.0 < measure ≤ 0.1, (ii) moderate: 0.1 < measure ≤ 0.42, (iii) high: 0.42 < measure ≤ 0.62, and (iv) very high: 0.62 < measure. Given that sharing a common indication or side effect element is more important in the occurrence of drug-drug similarity, we assumed that there is no information about the similarity of drugs when a value of a specific element at the vector was zero.

**Table 1:** Binary vector similarity measures

Measure	Equations	Description	Range
Jaccard	$S_{Jaccard} = \frac{a}{a + b + c}$	A normalization of inner product [23]	[0,1]
Dice	$S_{Dice - 2} = \frac{a}{2a + b + c}$	A normalization on inner product [24]	[0,1]
Tanimoto	$S_{{{\text{Tan}} imoto}} = \frac{a}{(a + b) + (a + c) - a}$	A normalization on inner product [22]	[0,1]
Ochiai	$S_{Ochiai - 1} = \frac{a}{{\sqrt {(a + b) + (a + c)} }}$	A normalization on inner product [25]	[0,1]

Suppose that two objects or patterns, i and j are represented by the binary feature vector form. a is the number of features where the values of i and j are both 1 (or presence), meaning 'positive matches', b is the number of attributes where the value of i and j is (0,1), meaning 'i absence mismatches', c is the number of attributes where the value of i and j is (1,0), meaning 'j absence mismatches'

**Fig. 2:**
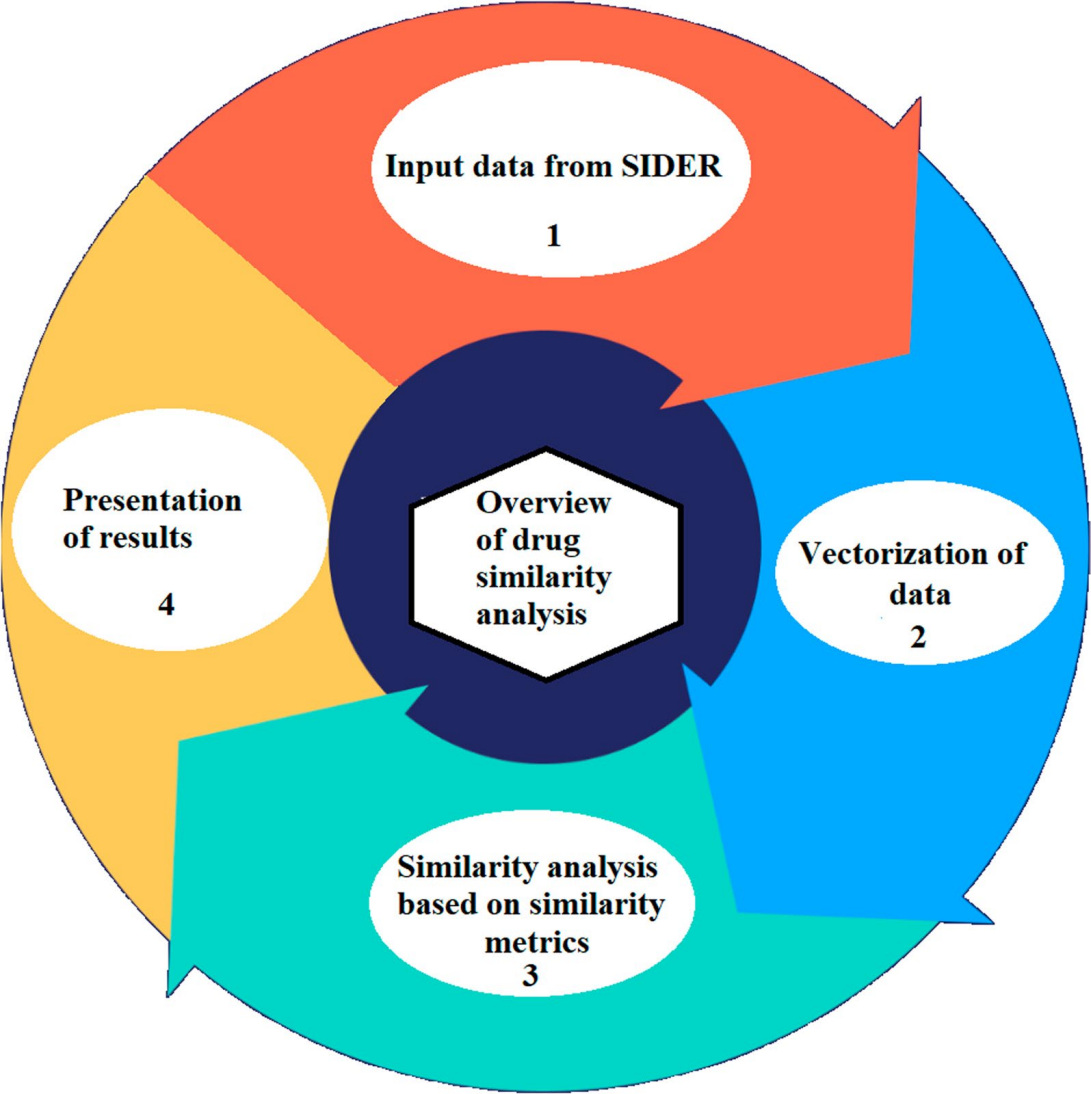
Overview of drug similarity analysis through side effects and indications

Analysis methods presented in this study could be developed using Windows or any other operating system with no special hardware requirements. Herein, we used Visual Basic and python Programming language for all computational and data preparation purposes. Excel 2016 and Pycharm software were utilized in our study to create different metrics, and data were interpreted using freely accessible Cytoscape 3.7.2 software. An overview of drug similarity analysis through side effects and indications is presented in Fig. 2.

## Results

**Fig. 3:**
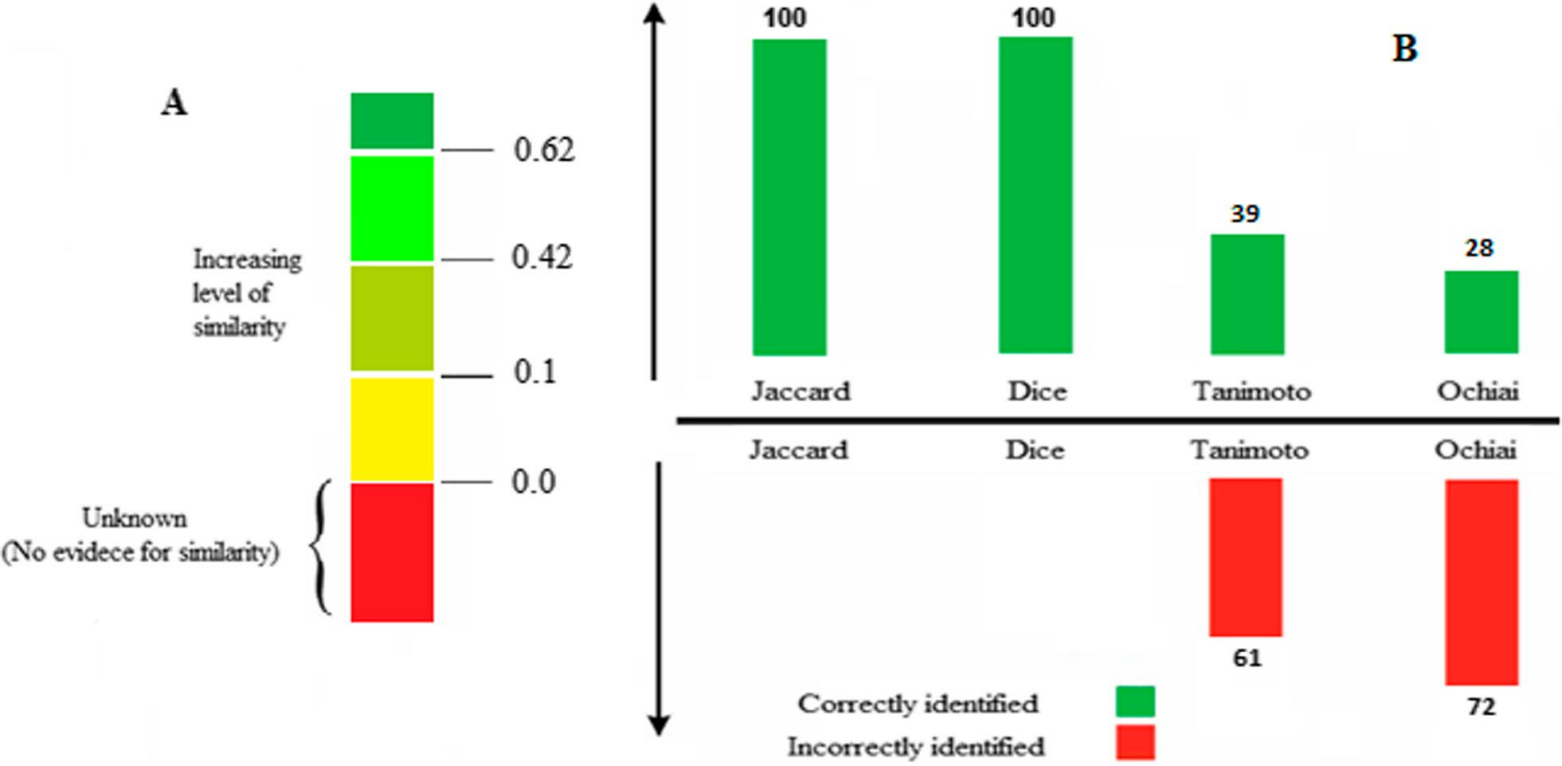
**A** Three split points are used as the threshold to categorize discovered drug pairs based on their level of similarity. Pairs with similarities lower than 0 are the pairs whose interaction possibility is low or unknown. **B**: Performance of the measures (the X-axis shows the similarity measures, and the Y-axis shows the performance of measures)

To determine which similarity measures fit best for detecting drug-drug similarity, all measurements were calculated, and different threshold points were considered for each step (Fig. 3A). To resolve the selection of similarity measure threshold, the lowest possible value for each measure was regarded as the minimum threshold. This allowed us to detect all potential Drug pairs even with low similarity or one common indication or side effect element shared.

A Study of similarity measures on drug-drug similarity vectors showed that Tanimoto and Ochiai measures failed to provide reliable similarity results because these methods consider similarity based on both negative and positive indexes (Fig. 3B). The Jaccard and Dice methods were found to fit better than the other ones (Fig. 3B). Finally, the Jaccard similarity measure was selected largely because of its precision and is easy to interpret through normalization between 0 and 1.

After selecting the Jaccard similarity method, it was applied for all drug pairs (i.e., 1,031,765 all possible drug pairs based on indications similarity and 4,489,506 all possible drug pairs based on side effects similarity).

**Fig. 4:**
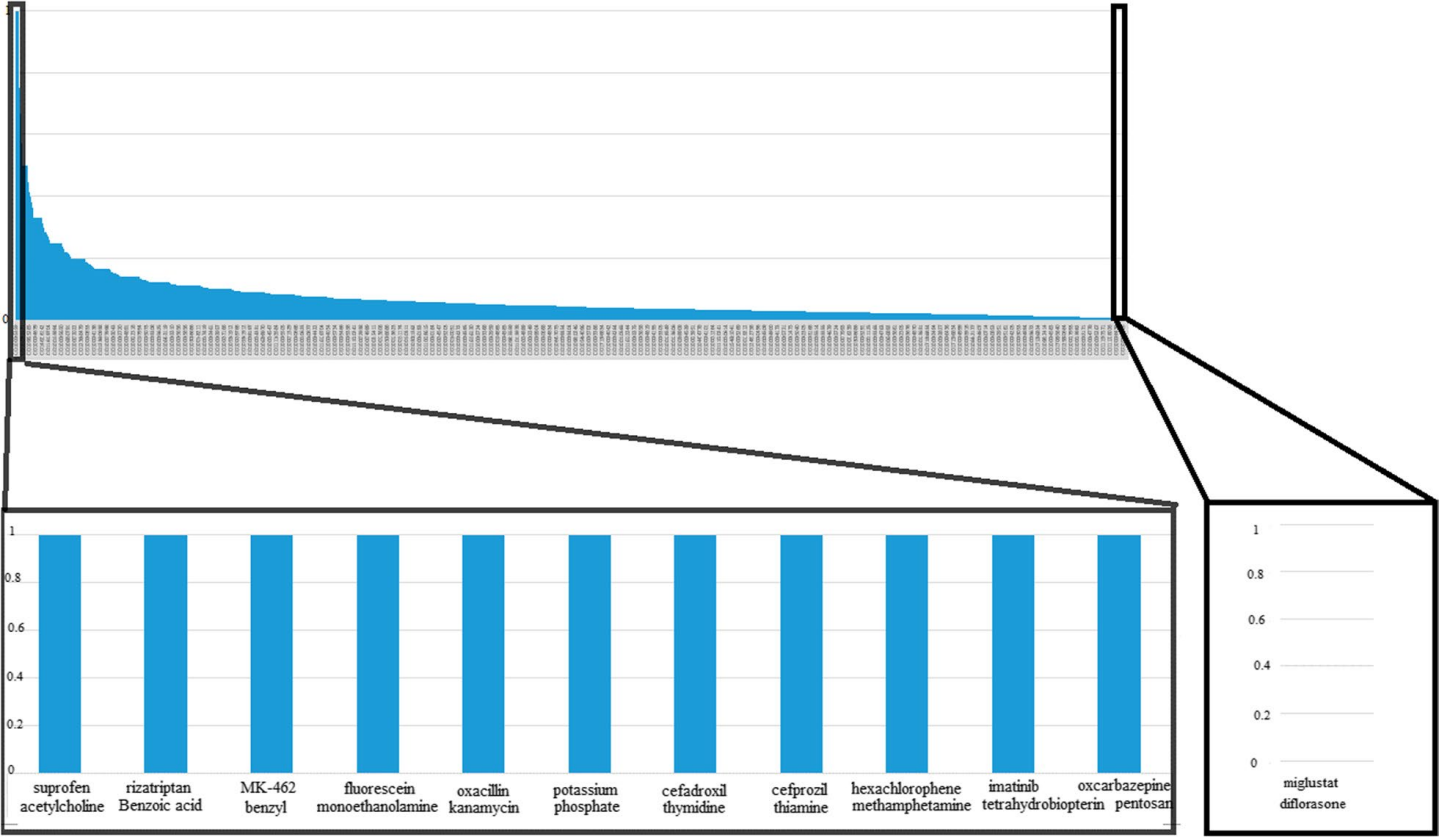
Ranked list of drug pairs based on their indications and similarity

**Fig. 5:**
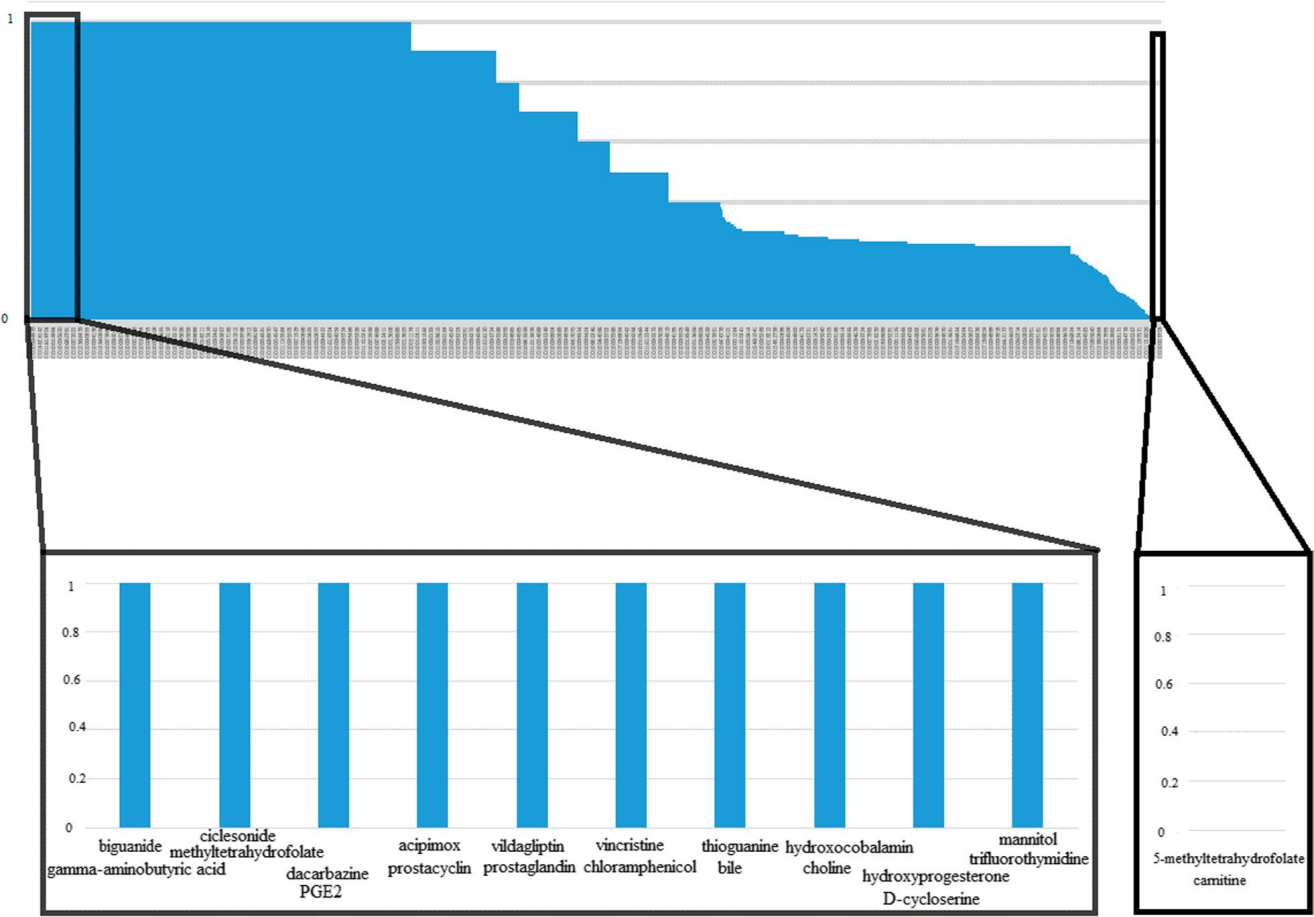
Ranked list of drug pairs based on their side effects and similarity

Having considered this measure, a threshold of similarity was set at zero. As can be seen in Figs. 4 and 5, the closer the result of the similarity measure is to one, the more similar the drugs are. That is, a large number of data elements related to indications or side effects are shared between similar drugs and the closer it is to zero, the greater the dissimilarity of the drugs.

For example, using this method, among known drug-drug similarities based on indication, rizatriptan benzoate (a medication used for the treatment of migraine headaches) and Benzoic acid (a drug used for the treatment of fungal skin diseases) were found to have about 28 common elements with the highest similarity level (1.0) (*p*-value = 0.015). Additionally, diflorasone (is used as an anti-inflammatory and anti-itching agent, like other topical corticosteroids) and miglustat (is a medication used to treat type I Gaucher disease.) were found to have about 0 common elements with the lowest similarity level (0.0) (*p*-value = 0.201) (Fig. 4(. Besides known drug-drug similarity based on side effects, Biguanide (a drugs group used for diabetes mellitus or prediabetes treatment) and gamma-aminobutyric acid (a drug used for reducing neuronal excitability throughout the nervous system) were found to have about 39 common elements with the highest similarity level (1.0) (*p*-value = 0.005). Also, carnitine (a drugs group that play a critical role in energy production) and 5-methyltetrahydrofolate (is the primary biologically active form of folate used at the cellular level for DNA reproduction, the cysteine cycle and the regulation of homocysteine) were found to have about 0 common elements with the lowest similarity level (0.0) (*p*-value = 0.105) (Fig. 5(.

**Fig. 6:**
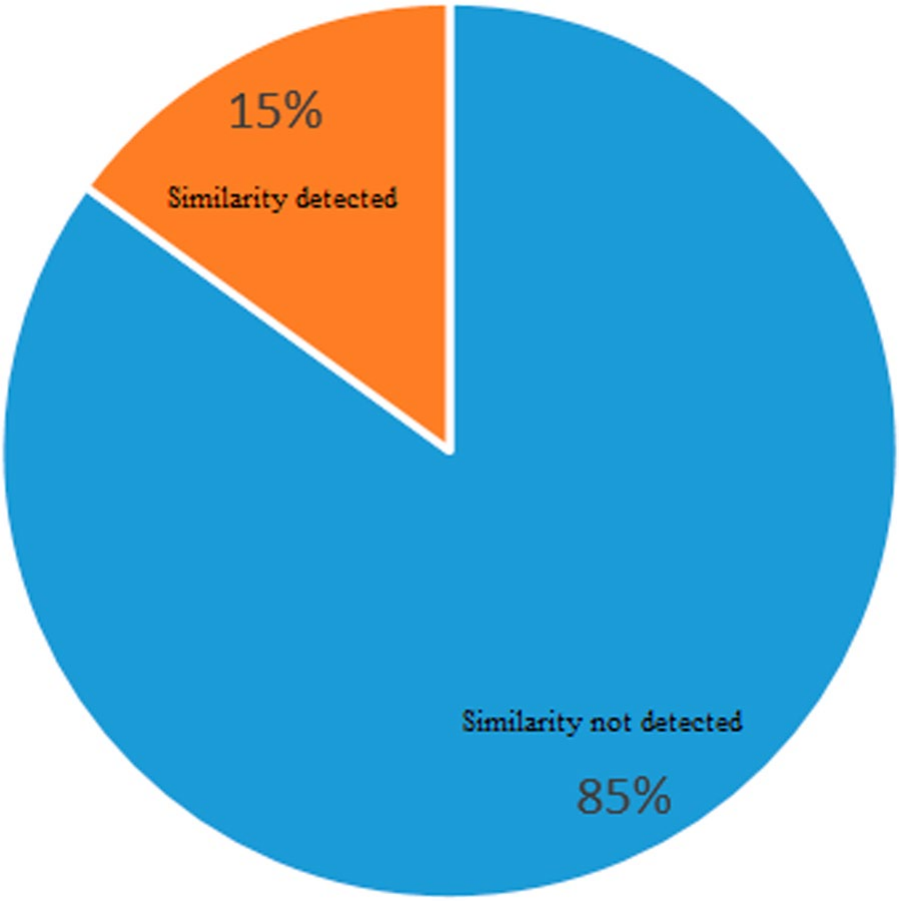
Observed Indications of similarity in drug pairs

**Fig. 7:**
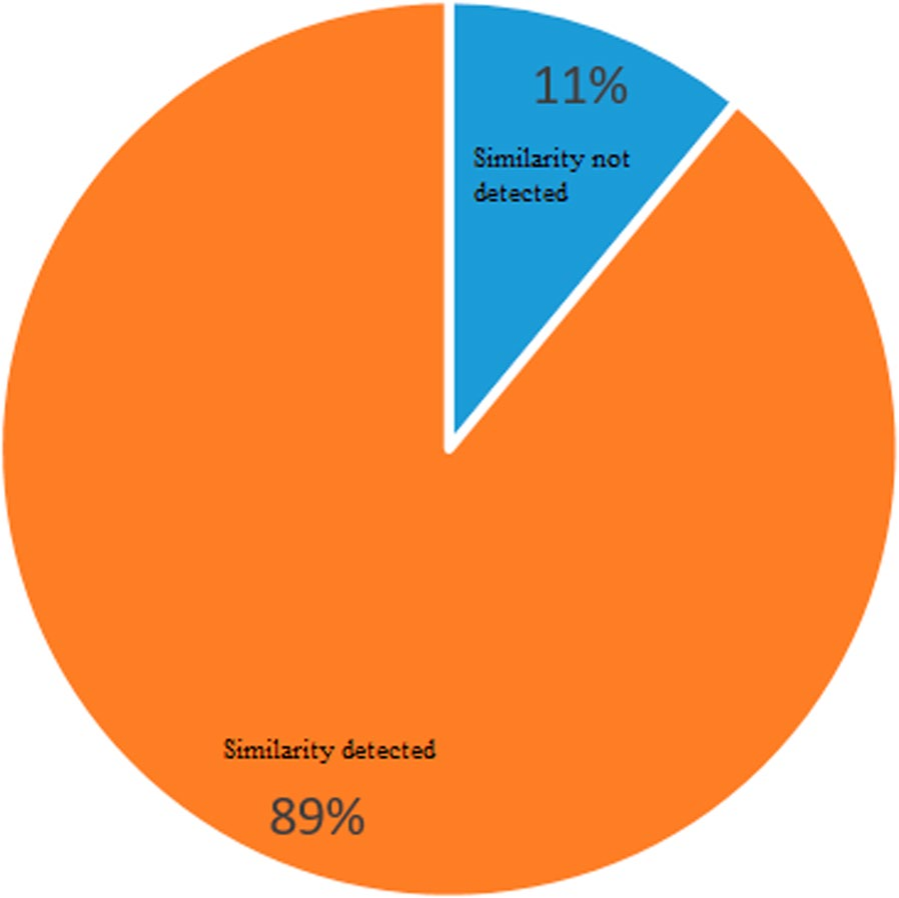
Observed Side effects similarity in drug pairs

After calculation of the similarity of all drug pairs and exclusion of empty vectors (vectors that all the elements' values are zero), 15% of the known drug-drug similarity were identified based on indication similarity, and 89% of the known drug-drug similarity were identified based on side effect similarity, in which there exist some shared indication and side effect between drug pairs. Accordingly, we assumed that side effect plays a central role in the occurrence of drug-drug similarity (Fig. 6 and Fig. 7).

To categorize the discovered drug-drug similarity based on their level of similarity, drug pairs were classified based on split points (Fig. 3A). Briefly, in drug pairs similarity based on their indications, 97% of discovered drug-drug similarity showed low level (103,088 pairs), 2.5% (2584 pairs) moderate level, about 0.2% (295 pairs) high level, and around 0.3% (307pairs) very high level of similarities. Moreover, in drug pairs similarity based on their side effects, 42.5% of discovered drug-drug similarity showed low level (1,635,233 pairs), 25% (959,957 pairs) moderate level, about 19% (729,856 pairs) high level, and around 13.5% (517,058 pairs) very high level of similarities (Table. 2).

**Table 2:** The number of identified pairs for each similarity level

Drug pairs similarity based on their indications		Drug pairs' similarity based on their side effects	
Level of similarity	Drug pairs	Level of similarity	Drug pairs
Low	103,088	Low	1,635,233
Moderate	2584	Moderate	959,957
High	295	High	729,856
Very high	307	Very high	517,058

As illustrated in Table 3, Hypertensive Disease, Myocardial Infarction, Angina Pectoris, Hyperlipidemia, Heart failure, Diabetic Nephropathy, and Diabetes Mellitus were found to be the shared indications elements between Felodipine and Aliskiren. Additionally, Table 4 shows that Anaphylactic shock, Angioedema, Urticaria, Injection site tenderness, Injection site pain, and Pain were found to be the shared side effect elements between gamma-aminobutyric acid and HBIG. Tables 3 and 4 represents eight more cases from identified drug-drug similarity, showing shared indication elements and side effect elements between drug pairs. Drug pairs with higher similarity scores share more indications or side effects.

**Table 3:** Examples of drug-drug similarity based on shared indication elements found by the Jaccard similarity method

Drug pairs	Shared indication elements	Similarity	*P*-value
Fluoxymesterone Testosterone	Wounds and Injuries, Malignant neoplasm of the breast, Hypogonadism, Neoplasms, Cryptorchidism, Orchitis, Puberty, Delayed Puberty, Testicular hypogonadism, Hypogonadotropic hypogonadism, Primary hypogonadism, and Testosterone deficiency	0.9166	0.016
Benazepril Benazeprilat	Hypertensive disease, Kidney Diseases, Renal Insufficient, Angioedema, and Renal Artery Stenosis	0.8544	0.021
Felodipine Aliskiren	Hypertensive disease, Myocardial Infarction, Angina Pectoris, Hyperlipidemia, Heart failure, Diabetic Nephropathy, and Diabetes Mellitus	0.75	0.028
Cortisol Methylprednisolone	Malignant Neoplasms, Edema, Pneumonia, Wounds and Injuries, Dermatologic disorders, Allergic conditions, Arthritis, Rheumatoid Arthritis, Dermatitis, Inflammation, Degenerative polyarthritis, Asthma, Diuresis, Hematological Disease, Tuberculosis, Hay fever, and so on	0.6950	0.032

**Table 4:** Examples of drug-drug similarity based on shared side effect elements found by the Jaccard similarity method

Drug pairs	Shared side effect elements	Similarity	*P*-value
Gamma-aminobutyric- acid HBIG	Anaphylactic shock, Angioedema, Urticaria, Injection site tenderness, Injection site pain and Pain	1.0	0.012
Estrone Estradiol-cyclopentylpropionate	Abdominal cramps, Depression, Dizziness, Anaphylactic shock, Rash, Dermatitis, Headache, Cramps of lower extremities, Muscle spasms, Nausea, Pruritus, Musculoskeletal discomfort, Anaphylactic shock, Urticaria, etc	0.9733	0.019
Flumethasone Alclometasone	Secondary infection, Dermatitis, Pruritus, Leukoderma, Folliculitis, Allergic contact dermatitis, Skin atrophy, Acneiform eruption, and Dermatitis perioral	0.8235	0.025
Alclometasone Clocortolon	Rash, Dermatitis, Leukoderma, Folliculitis, Allergic contact dermatitis, Skin striae, Pruritus, and Eruption	0.7222	0.041

Linear regression was used to inspect the association between drug similarity based on indications and drug similarity based on side effects. Linear regression results have revealed a significant relationship between drug similarity based on indications and drug similarity based on side effects (*p*-value = 0.03). This means that the similar drugs, based on indications, are similar (based on the side effects).

**Fig. 8:**
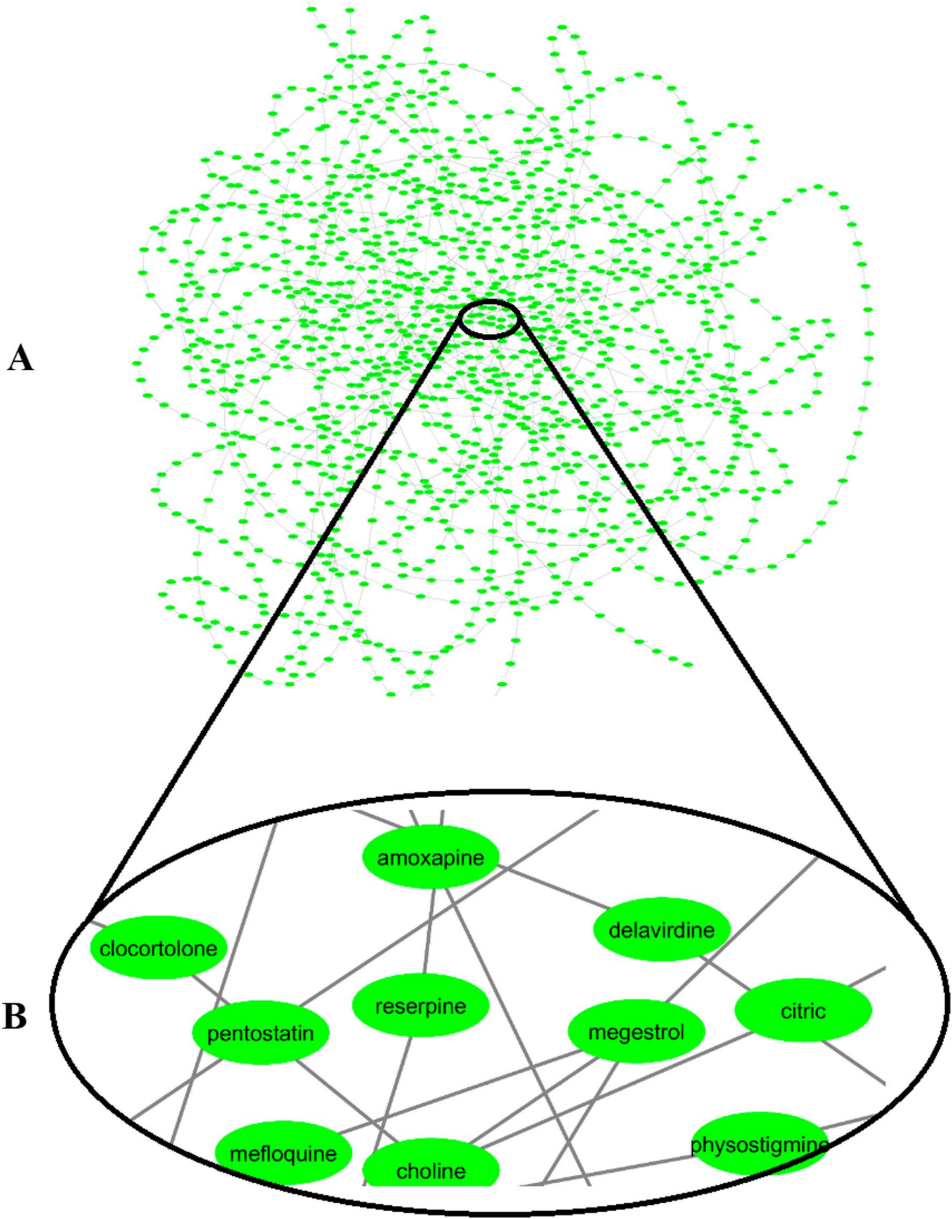
Drug similarity network based on the indications similarity (panel **A**), a sub network of drug similarity network based on the indications similarity (panel **B**)

**Fig. 9:**
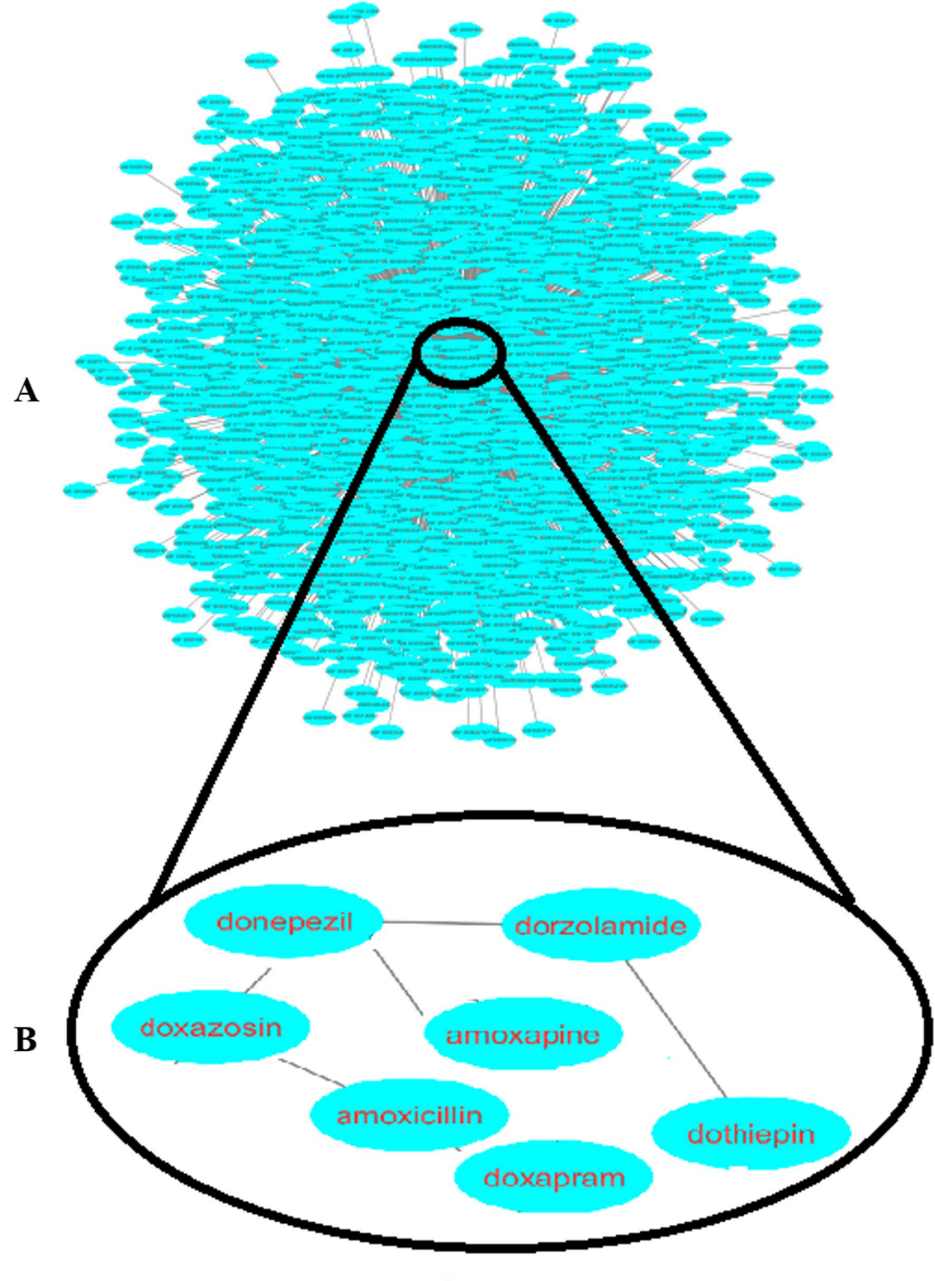
Drug similarity network based on the side effects similarity (panel **A**), a sub network of drug similarity network based on the side effects similarity (panel **B**)

Finally, a network was formed based on the similarities found between drug pairs for understanding drugs relationships. As Figs. 8 and 9 represents in this network, the most similar drugs were closely linked and central to the network. Furthermore, as the similarity between the drug pairs declines gradually, the connections become farther and more peripheral. Because the weight of connections between drug nodes in the network is determined based on the number of common indication or side effects data elements between drug nodes. As a result, the more drugs are similar to each other, the weight of the connections will be stronger, and as a result, the nodes will be closer to each other and will build a centralized network.It also exhibits that drugs' similarity based on side effects is much more than the similarity of indications-based drugs.

## Discussion

The detection of drug-drug similarity is one of the most vital matters in pharmacotherapy performance. Effective pharmacotherapy and administration are essentially dependent upon the identification of potential drug-drug similarity [4]. Recently, there has been growing interest in predicting drug-drug similarities, which can have multiple potential applications, such as the prediction of novel drug-drug interactions. The main idea of the similarity-based approach is to predict it by comparing the presence of similarity between a pair of subjects. However, present similarity-based methodologies are difficult to distinguish between low similarity values. Moreover, these existing drug similarity measurement techniques rely on a limited number of data sources that can merely provide partial information about a subset of drugs of interest, leading to varying levels of incompetency [26–30].

Diverse methods of estimating drug similarity have various scenarios and advantages for use. Chemical similarity, for example, plays an imperative role in predicting the properties of chemical compounds, identifying the underlying drug interaction, and performing drug design studies especially. Nevertheless, only a few clinical drugs are single chemical substances, many of which are bio-pharmaceutical or compound medicine, lacking chemical structure data [31].

In the current study, we have developed binary vectors for predicting drug-drug similarity based on indications and side effects elements. Besides, identifying potential drug-drug similarity for all drugs requires rationalized computational analysis (e.g., similarity measures as listed in Table 1). We used four different similarity measures to select a reliable universal drug-drug similarity prediction method. We picked up the Jaccard method as an accessible, straightforward, precise, consistent, interpretable, and scalable method.

The Jaccard method provides an interpretable and simple similarity measure between 0 and 1 (Fig. 3B). It should be emphasized that this method is sensitive to positive matches and only differ in range. The similarity trends of drug pairs were evaluated to determine the potential similarity of drugs based on indications and side effects. To be much more precise in drug-drug similarity prediction, the performance of all measures (Table 2) was compared to each other based on the similarity values. In the case of drug-drug similarity, the negative indexes do not unavoidably reflect the similarity. In a vector, the number of elements with an index value of 0, as compared to those with the index value of 1, is considerably high in most cases. Thus, if a similarity measure considers the negative matches that are substantially huge, the indication or side effect elements-oriented vectors for prediction of drug-drug similarity may result in significantly high similarity that could reflect false similarities.

To the best of our knowledge, this current study is the first investigation that utilizes the potential of the Jaccard for the prediction of drug-drug similarity. For the validity of the current investigation, three split points were used to categorize the detected drug-drug similarity based on their level of similarity. The pairs with similarities lower than 0 were considered to have a low value or an unknown phenomenon (Fig. 3A). In this study, 5,521,272 drug pairs were analyzed, which resulted in detecting over 3,948,378 new possible drug-drug similarities. The discovered drug-drug similarity was categorized based on their similarity. This information can have used for providing medical recommendations and rational drug design and development. Similarity measures are the foundation of all modern pattern classification and clustering algorithms. Similarity has massively been utilized in different fields such as image retrieval, information retrieval, chemistry, ecology, psychology, and biology [4].

The methods currently used to discover drug-drug similarity focus on gathering sufficient clinical evidence. Nonetheless, in this technique, drug-drug similarity can be identified through computational procedures. These cost-effective solutions are critical not only for the pharmaceutical industry but also for health care providers. Pharmaceutical corporations can forecast potential drug-drug similarity and use such information to advance new drugs, refine product formulations, and provide customers with the necessary information [32, 33]. We just used the Side Effect Resource (SIDER 4.1) database as the fundamental resource, largely because of the large size of data existing in SIDER 4.1. In fact, we used this source for the compatibility and consistency of information. SIDER is a comprehensive resource for adverse drug reactions and indications extracted from drug labels and other resources. However, it has not been updated since 2015. It is suggested that future studies utilize information from multiple sources that are regularly updated to ensure that up-to-date data and new drugs are considered.

There are several resources for drug-drug similarity in the literature. Jin et al*.* presented a summarization of accessible drug-drug similarity using molecular structure data [34]. Cheng and Zhao computed drug similarity using side-effect information [35]. Besides, Fokoue et al*.* provided drug similarity based on the interaction profile data [19].

Several studies denoted good performance in which the drug-drug similarity prediction used machine learning algorithms. Nevertheless, it is almost impossible or difficult to interpret the origin of the resemblance incidence. For instance, despite the reliable performance of the support vector machine technique, casualty interpretation and reasoning of the occurrence of drug-drug similarity appear to be challenging problems. Another problem regarding such algorithms is the preparation process of the negative set [36]. The current study provides clear evidence for the known similarity (shared indication and side effect elements) and presents the reasons for the expected drug-drug similarity. Additionally, the drug-drug similarity pairs described in this study have at least one specific indication or side-effect element between two drugs.

The drug similarity can be adopted to quantitatively measure the similarity of medical therapy and further patient similarity, which is an evolving notion in systems and precision medicine. Patient similarity investigates distances between varieties of components of patient data and determines methods of clustering patients based on short distances between some of their characteristics [37]. Among the similarity, which means a group of similar patients, index patients can be evaluated through further stratification driven by individual diagnosis, risk factors, medication, etc. So far, several algorithms have been advanced to estimate the different types of clinical data, such as diagnosis and the laboratory test outcome. However, it isn't well known how to measure the similarity in drug therapy [31].

The limitation of the methodology in this paper is that, unlike other computational methods that utilize molecular structure, for example, to measure drug-drug similarity, this method cannot be applied to investigational drugs and drugs with unknown drug safety profiles side effects and indications.

## Conclusion

This study was conducted to focus on high-throughput statistical exploratory approaches to drug-drug similarity prediction based on the indications and side effects. We employed four different similarity measures for selecting a reliable universal drug-drug similarity prediction method. We opted for the Jaccard method, largely due to its simplicity and applicability. We envisage that this method, which is a standardization of the approach to the inner product, can serve as a reliable method for drug-drug similarity prediction. We recommend this approach to large volumes of data as an accessible, precise, consistent, interpretable, and scalable method. Our findings revealed similarity of 106,274 drug pairs based on indications and similarity of 3,842,104 drug pairs based on side effects as new possible drug-drug similarity, which is a good sign for the validity of this approach, yet each pair of drugs with latent similarity detected in this study may need to be validated through in vitro and/or in vivo experiments.

We assume that categorizing the observed drug-drug similarity dependent on their similarity values can be in favor of patient clinical trials and hence safer pharmacotherapy. The results of this study can also provide a forum for further in vitro and in vivo exploratory and confirmatory testing-important for patient care. Additionally, we assume that molecular structure or disease-related data may be applied the evidence to make more comprehensive and precise interpretations of drug-drug similarity phenomena. This method could be a contributing factor in the success of care modalities. The approach provided for recognizing drug-drug similarity is flexible and could apply to broad volumes of data based on the indication or side effect elements between drugs. Moreover, considering personalized information on the functional expression of indication or side effect elements for each individual, the computational application can be built soon for healthcare providers and patients to track drug-drug similarity.

## Data Availability

The datasets supporting the conclusions of this article are available in the SIDER 4.1 repository. (http://sideeffects.embl.de/download/).
